# The Impact of Growth Mindset on Older Adults’ Cognitive Functioning in a Multi-Skill Learning Intervention

**DOI:** 10.1093/geroni/igab046.1200

**Published:** 2021-12-17

**Authors:** Pamela Sheffler, Esra Kürüm, Angelica Sheen, Leah Ferguson, Diamond Bravo, George Rebok, Carla Strickland-Hughes, Rachel Wu

**Affiliations:** 1 University of California, Riverside, Riverside, California, United States; 2 University of California, Irvine, Irvine, California, United States; 3 Johns Hopkins University, Baltimore, Maryland, United States; 4 University of the Pacific, University of the Pacific, California, United States; 5 University Of California - Riverside, Riverside, California, United States

## Abstract

Motivational factors, such as perceived control and self-efficacy, have been shown to affect older adults’ cognitive functioning. Growth mindset, the belief in the malleability of intelligence and abilities, represents a related but distinct factor that has been widely studied in children and young adults’ learning but less applied to the older adult population. Two studies investigated growth mindset, motivation, and cognitive functioning in a 3-month multi-skill learning intervention that incorporated weekly discussions on growth mindset and successful aging. Participants reported on their growth mindset, general pursuit of novel skill learning, and intrinsic motivation to learn, and completed a cognitive battery before, during, and after the intervention. Study 1 (n = 15, 67% female, M age = 68.67 years, SD age = 8.68, range 58-86) included both an experimental and control group and indicated that from pretest to post-test, intervention participants increased their growth mindset, while control participants did not. Study 2, which included a larger, all experimental sample (n = 28, 68% female, M age = 69.36 years, SD age = 7.00, range 58-86) revealed strong positive associations between growth mindset, pursuit of novel skill learning and intrinsic motivation. Further, participants showed a significant increase in growth mindset from pretest to post-test. Participants with higher pre-existing growth mindset showed larger cognitive gains at post-test, although growth mindset change did not affect post-test change in cognitive functioning. These findings suggest that growth mindset may facilitate older adults’ continued learning and cognitive gains, and they may complement older adult learning interventions.

